# Camels’ biological fluids contained nanobodies: promising avenue in cancer therapy

**DOI:** 10.1186/s12935-022-02696-7

**Published:** 2022-09-07

**Authors:** Nouf S. Al-Numair, Abdulrahman Theyab, Faisal Alzahrani, Anwar M. Shams, Ibrahim O. Al-Anazi, Atif Abdulwahab A. Oyouni, Osama M. Al-Amer, Charalampos Mavromatis, Islam M. Saadeldin, Wed A. Abdali, Yousef M. Hawsawi

**Affiliations:** 1 Center of Genomic Medicine, King Faisal Specialist Hospital & Research Center, Riyadh, Saudi Arabia; 2 College of Medicine, Alfaisal University, P.O. Box 50927, Riyadh, 11533 Saudi Arabia; 3 Department of Laboratory & Blood Bank, Security Forces Hospital, P.O. Box 14799, Mecca, 21955 Saudi Arabia; 4 College of Medicine, Al-Faisal University, P.O. Box 50927, Riyadh, 11533 Saudi Arabia; 5 Department of Biochemistry, Faculty of Science, Embryonic Stem Cells Unit, King Fahad Medical Center, King Abdulaziz University, Jeddah, Saudi Arabia; 6 Centre of Artificial Intelligence in Precision Medicines (CAIPM), King Abdulaziz University, Jeddah, Saudi Arabia; 7 Department of Pharmacology, College of Medicine, Taif University, P.O. BOX 11099, Taif, 21944 Saudi Arabia; 8 The National Center for Genomic Technology, King Abdulaziz City for Science and Technology, P.O Box 6086, Riyadh, 11442 Saudi Arabia; 9 Department of Biology, Faculty of Sciences, University of Tabuk, Tabuk, Saudi Arabia; 10 Genome and Biotechnology Unit, Faculty of Sciences, University of Tabuk, Tabuk, Saudi Arabia; 11 Department of Medical Laboratory Technology, Faculty of Applied Medical Sciences, University of Tabuk, Tabuk, Saudi Arabia; 12 Department of Biological Sciences, Faculty of Science and Arts (Rabigh Campus), King Abdulaziz University, Jeddah, Saudi Arabia; 13 Research Institute of Veterinary Medicine, Chungam National University, Daejeon, 34134 Korea; 14 Research Center, King Faisal Specialist Hospital and Research Center, MBC-J04, PO Box 40047, Jeddah, 21499 Saudi Arabia

**Keywords:** Nanobodies, Arabian camelid, Biological fluids, Cancers, Therapeutic agents, Diagnosis, And nano proteomics

## Abstract

Cancer is a major health concern and accounts for one of the main causes of death worldwide. Innovative strategies are needed to aid in the diagnosis and treatment of different types of cancers. Recently, there has been an evolving interest in utilizing nanobodies of camel origin as therapeutic tools against cancer. Nanotechnology uses nanobodies an emerging attractive field that provides promises to researchers in advancing different scientific sectors including medicine and oncology. Nanobodies are characteristically small-sized biologics featured with the ability for deep tissue penetration and dissemination and harbour high stability at high pH and temperatures. The current review highlights the potential use of nanobodies that are naturally secreted in camels’ biological fluids, both milk and urine, in the development of nanotechnology-based therapy for treating different typesQuery of cancers and other diseases. Moreover, the role of nano proteomics in the invention of novel therapeutic agents specifically used for cancer intervention is also illustrated.

## Cancer and nanobodies at a glance

Cancer denotes one of the leading causes of death globally albeit with the rapid innovations in the molecular biology [1]. According to statistics produced by the GLOBOCAN series of the International Agency for Research on Cancer (IARC), over 14.1 million cancer cases and more than 8.2 million cancer-related deaths were reported in 2012 [2]. Approximately, 21.7 million cancer cases and over 13 million cancer-related deaths are predicted to occur by 2030 [1]. Successful interventions of cancer progression in affected patients are attributed to early detection and prompt implementation of the treatment plan which often involves surgery, radiation, chemotherapy, targeted therapy, and hormone therapy [3]. Following decades of research, researchers have now begun to explore nanotechnology-based therapy using nanobodies and evaluating their potential uses as novel cancer therapeutic modalities. Nanobodies can cross the blood–brain barrier and invade large solid tumours more competently than other conventional antibodies. This encourages experts to propose nanobodies as ideal therapeutic candidates for the solid metastatic cancers [4].

## Distinguished features of the nanobodies and nanoparticles

Small molecules, or nanoparticles, have a diameter of roughly 200 nm. Many pharmaceutical companies have aspired to create efficient drug delivery methods, and as a result, a variety of nanoparticle-based delivery modalities, including magnetic, polymeric, and inorganic nanoparticles, have been developed [5]. An attachment of the targeting moieties to the drug cargo has been devised to improve the transport, permeability, and penetration of the drug-conjugated nanoparticles into the target tissues. Poly-ethylene glycol (PEG) molecules and nanobodies are examples of these targeted molecules that protect the nanoparticles [6]. Decorating nanoparticles with nanobodies enhanced the accumulation of the nanoparticles containing the drug’s cargo into the diseased tissues [7]. By monomerizing the dimeric variable domains of human or mouse conventional antibodies, nanobodies can be bioengineered. Nanobodies, with a molecular mass of roughly 15 kDa, are thought to be the smallest antibody components capable of antigen detection [8]. Alternatively, nanobodies can also be retrieved and isolated from the immunized camel blood and characterized as a single variable domain on a heavy chain (VHH) antibodies/nanobodies [6, 8]. VHH, or Heavy chain antibodies (HCAbs), that are derived from camel blood have lower lipophilicity and are a single domain consisting of one amino acid chain. These characteristics confer certain advantages to VHH over conventional antibodies which generally consist of two amino acid chains and are 10 × larger in size (approximately 150 kDa).

**Fig. 1:**
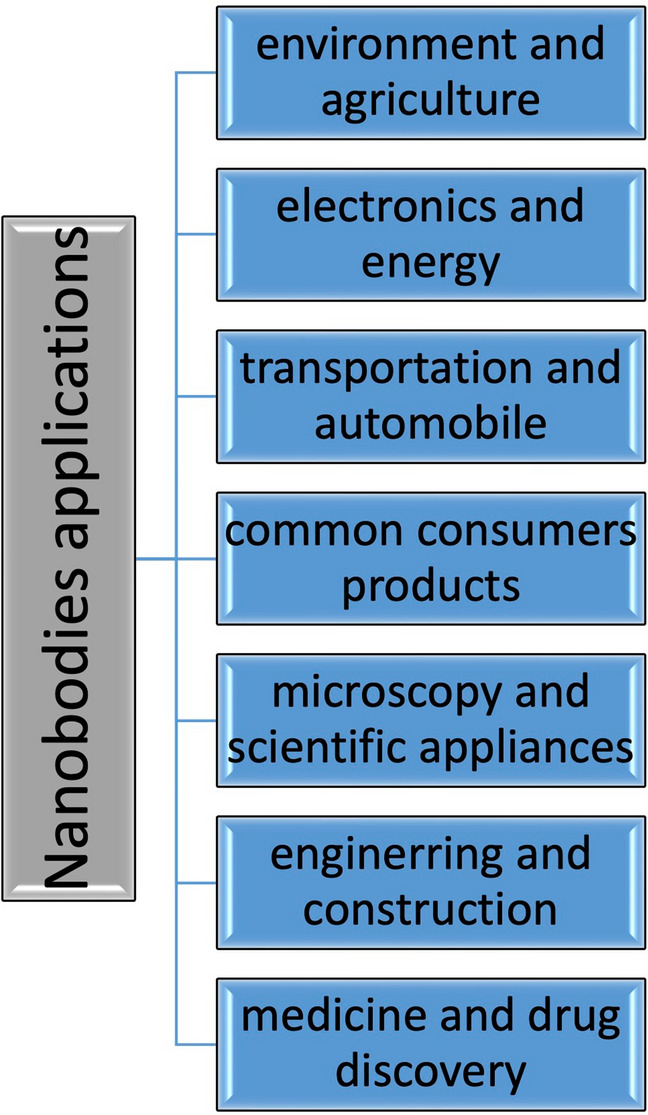
Nanobodies applications

Nanobodies exemplify unique structural and functional features credited to their small size (15–74 kDa) and large surface area, high stability and solubility, high binding affinity and detection of different epitopes, fast tissue internalization, ease of production and manipulation, and low immunogenic reactions [21]. Consequently, as shown in Fig. 1, nanobodies were engaged in numerous scientific and medical sectors encompassing environment and agriculture, electronics and energy, transportation and automobile, common consumer products, microscopy and scientific appliances, engineering and construction, and medicine and drugs discovery [22]. The implication of bio-engineered nanobodies as novel therapeutic tools for many diseases has been substantiated in several studies. For instance, nanobodies directed against hemagglutinin influenza A H5N1 have been shown to suppress virus replication in infected mice and reduce both morbidity and mortality [23]. Nanobodies target the binding area of virulence factors, such as toxin A and toxin B of Clostridium difficile and neutralize cytopathic effects in fibroblasts in vitro [24]. Gastrointestinal tract syndromes, including colon cancer and inflammatory bowel disease, were also reported to be effectively targeted by nanobodies [16]. Other remarkable nanobodies-based therapies include ALX-0081 and viral-based cancer therapeutics packaged with nanobodies. In a clinical trial, ALX-0081 was successfully used as a target for the Von Willebrand factor to prevent the risk of thrombosis in patients with acute coronary syndrome [4]. Nanobodies have also been used in photothermal therapy due to their ability to bind tumour antigens, like HER2, such as cleft-gold nanoparticles that absorb light energy to generate heat that destroys cancer cells. Thus, malignant cells can be destroyed photothermally after exposure to a laser beam in an experimental environment [25]. Moreover, due to the advent of viral-based cancer therapies that can be integrated with most cells whilst only replicating within cancer cells, packaging the viral vectors with nanobodies would ensure tumour-specific targeting. This would be a valid tool in targeting metastatic cancer effectively [26].

## Nanobodies and nanoparticle-based cancer therapy

Targeted delivery is a major milestone in cancer therapy as it mitigates the damage caused by non-selective drugs. Nanoparticles allow a controlled release of drugs inside the cancer cells using multi-functional nanocarriers. Consequently, the therapeutic potential of nanobodies as tools to deliver drug-loaded nanoparticles toward tumours has recently attracted the attention of many researchers [27, 28]. The nanoparticle-based approach concentrates the drug within target cancerous cells and protects the surrounding healthy cells and tissues from cytotoxic agents. Several researchers have developed modified nanoparticles for use as a potential target for anticancer drugs with success [29]. For example, nanoparticles have been successfully conjugated with nanobodies that express high specificity for the cancer marker Mucin-1 [30]. Mucin-1 overexpression is related to breast and colon cancers [31]. Nanoparticles made from polymers have been designed to carry a “killer gene”, which causes cell death when expressed in the target cells. The expression of the killer gene was controlled by the Mucin-1 promotor gene [30]. This specification allows the killing of only cancerous cells and shields healthy tissue from the toxicities associated with cancer treatment.

## Arabian camel’s genome and Nanobodies emergence

**Fig. 2:**
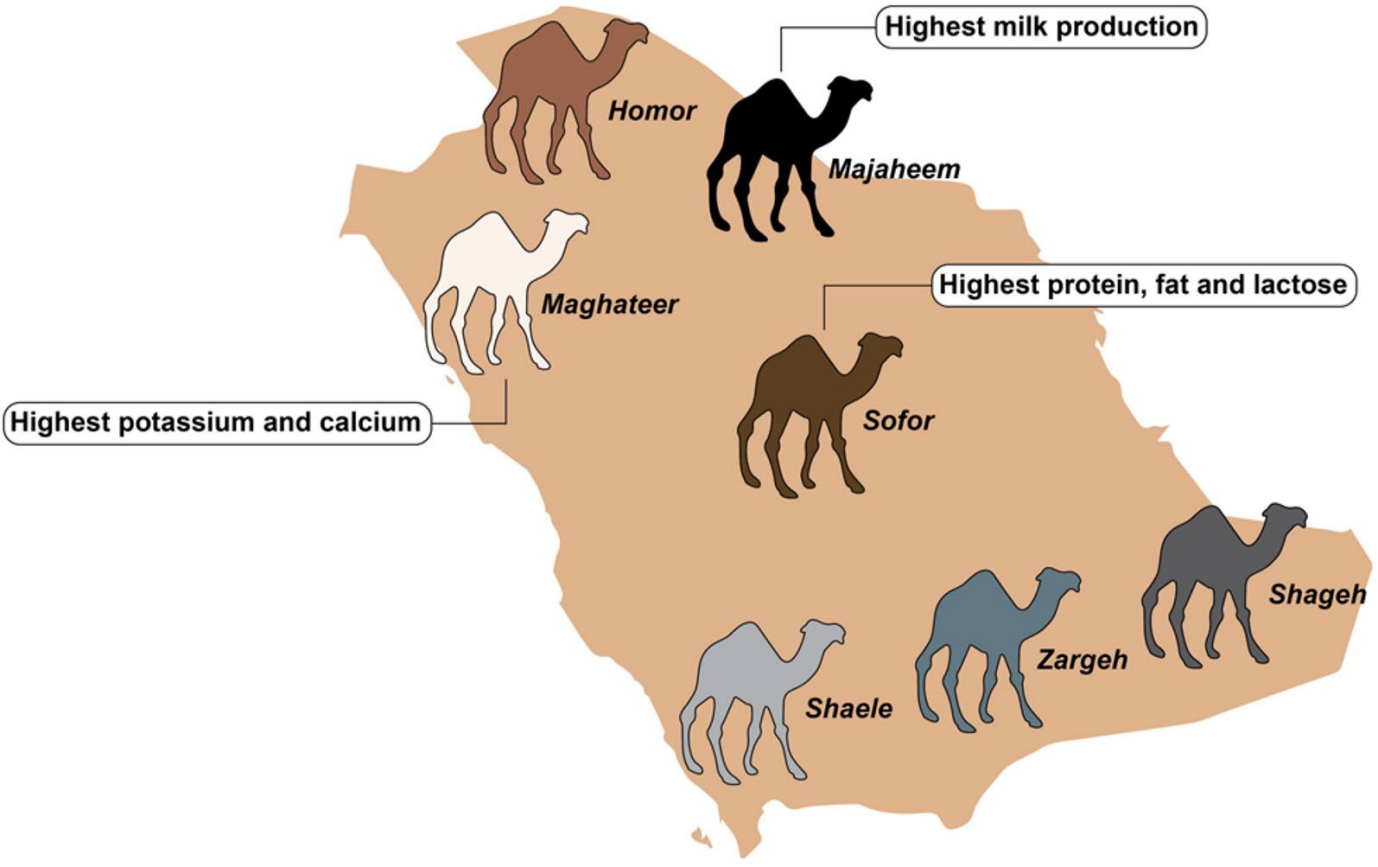
The diversity and distribution of Camels in Saudi Arabia

There are two surviving species of camels: Camelus dromedarius and Camelus bactrianus with each having distinguishing features. Camelus dromedarius (C. dromedarius) also called the dromedary, one-humped camel, or Arabian camel mainly lives in the hot deserts of North African and Middle Eastern countries, including Saudi Arabia (SA) [32, 33]. Whereas the Camelus bactrianus (C. bactrianus), also known as the Bactrian, or two-humped camel, lives in the cold desert of Asia. The C. dromedarius exhibits various unique traits, for instance, it can survive and reproduce in the extreme heat and drought conditions of the desert. In 2010, there were more than 830,000 different dromedary breeds in SA. This number significantly increased to 3,113,628 in 2015. Additionally, camels in SA are classified into seven different C. dromedarius breeds according to their coat colour: Majaheem, Maghateer, Homor, Shaele, Zargeh, Shageh, and Sofor, as demonstrated in Fig. 2 [32, 33].

The genome of the C. dromedarius camel has been completely sequenced as a result of the joint efforts of researchers from the Beijing Genomics Institute (BGI), China and the National Center for Genomic Technology (NCGT) at King Abdulaziz City for Science and Technology (KACST), Saudi Arabia [34]. Sequencing the complete genome is considered a breakthrough and a vital achievement in understanding the distinctive traits of this mammal. Availability of the genomic data is particularly essential to gaining novel insights into the molecular mechanisms underlying the production and secretion of nanobodies in the biological fluids [35]. Nanobodies were reported in camels in the early 1980s, the presence of which makes camels unique. Importantly, camel milk contains nanobodies that potentially can be used as a promising tool for diagnosing and targeting metastatic cancers [36–39].

### Camel-based nanobodies

The fact that all camelids naturally produce different antibodies that circulate in their blood should be kept in mind. Two identical heavy chains and two identical light chains joined by disulphide bonds through non-covalent interactions make up the usual structure for conventional antibodies [40]. On the contrary, as implied by their name, the heavy chain antibodies (HCAbs) are distinguished molecules that display unique features by having a heavy-chain homodimer while lacking light chains [16]. In contrast to traditional antibodies, these HCAbs are simple to isolate and clone from the serum of an immunized camelid and show lesser immunogenic reactivity [8]. In C. dromedarius (dromedary) the HCAbs are devoid of the standard first constant domain (CH1) and instead possess a single antigen binding site for which they called the variable domain of heavy chain of HCAbs (VHH) or nanobodies [35, 41]. The protein-engineering strategy has used a single-domain unit approach to enhance the efficacy of the large size conventional antibodies. Besides several biological and economic properties, the single-domain binding-based antibodies showed an endogenous agonistic or antagonistic effect on their target cells. In comparison to conventional antibodies, nanobodies, with single-domain moiety, provided a unique therapeutic avenue by exhibiting smaller size, are costless and easier to make, and higher solubility and heat stability [17, 18]. Likewise, single-domain antibodies are more resistant to gastric acid due to having a smaller number of cleavage sites and are more resistant to proteolytic enzymes than conventional antibodies [19]. This resistance can be increased by further optimizing the structure of single-domain antibodies to make them more permeable to the gastrointestinal tract and thus suitable for local per-oral applications [17]. Furthermore, as their molecular mass is below the renal threshold, single-domain antibodies have a very short plasma retention time and can be excreted via the kidneys into the urine [20]. They also show less cytotoxicity due to the absence of the FC fragment, which revealed a role in the activation of the complement system. Additionally, high specificity and affinity to the targeted antigen in combination with rapid cellular internalization allow the nanobodies to work faster and exhibit a superior efficacy over the conventional antibodies [19, 42]. Interestingly, the single-domain antigen-binding property exhibited by camelids VHH was utilized to generate various antibody constructs (bivalent, biparatopic or bispecific constructs) to enhance their specificity and affinity to their target antigens, or to bind two individual antigen particles [43]. This approach was applied to produce vastly stable antibody constructs recognizing clinically significant human prostate-specific antigen (hPSA) within only 15 min [44]. Similarly, camelid VHH presented robust effects as a talented tool that can be potentially implanted in several biotechnological and medical fields. For instance, cAbBCII10 is a specific VHH framework that has the ability for transferring the antigen specificity from the donor VHH onto its scaffolds following the grafting complementarity determining region (CDR) technique [45].

Development of a therapeutic tool targeting selectively the tumour cells and leaving the normal cells unaffected is a challenging aim. Cancer cells are featured by overexpression of specific receptors or biomarkers that can act as an attractive spot to increase the delivery and the accumulation of the drug into the affected tissues [25]. Following a ligand-receptor or antibody-antigen-based approach, conventional monoclonal antibodies have been widely used in biomedical research and clinical medicine. Accordingly, numerous vehicles for carrying drugs to their therapeutic destination for treating various malignancies have been constructed [6, 46]. These involve arcitumomab (anti-carcinoembryonic antigen (CEA)), capromab (anti-prostate-specific membrane antigen (PSMA)), trastuzumab (anti-human epidermal growth factor receptor 2 (HER2)), bevacizumab (anti-vascular endothelial growth factor (VEGF)), and cetuximab (anti-epidermal growth factor receptor (EGFR)) antibodies [6, 47, 48]. Although the use of traditional nanoparticle or murine monoclonal antibody therapy in clinics has produced positive outcomes, there are still a number of drawbacks. [9–12]. These include their fragile and complex structure that requires higher expenses during the production [46]. Additionally, it was shown that murine antibodies induced an immunogenic response by generating human anti-mouse antibodies, which led to their neutralization and loss of potency. Additionally, typical antibodies with a large molecular weight (approximately 150 kDa) have limited tissue penetration, extravasation, and permeability, which results in a heterogeneous distribution of the medication within the intended tissue [13]. Likewise, conventional nanoparticles exhibited a slower diffusion pattern and pose a significant hindrance in the drug release to the tumours which is associated with the and suboptimal therapeutic effects [14, 15]. Accordingly, tumour cells don’t expose to similar concentrations of the drugs and further this concentration doesn’t reach the therapeutic level that ensued cellular death resulting in reducing treatment efficacy [11–13]. As a result, many cancer patients exhibited resistance to antibody-based approach, which was linked to therapy failure and poor results. It is advised that the size of these nanoparticles should be between 5 and 200 nm in order to increase the effectiveness of the antibody-based therapy. In fact, nanoparticles less than 5 nm can be removed from the bloodstream with ease, whereas those larger than 200 nm will get caught in organs with reticuloendothelial systems, like the liver and spleen. Consequently, it was recommended that the ideal size of the utilized nanoparticles be less than 100 nm and have a hydrophilic capacity [6, 49]. The HCAbs camelid antibodies revealed a molecular mass of around 95 kDa and the VHH domain showed about 12–14 kDa mass thus they are considered as the minute intact antigen-binding entities that are naturally occurring for that named nanobodies [8]. Therefore the current trend in the establishment of drug delivery has switched to the use of smaller molecules with high specificities, such as camelid nanobodies, to overcome the existing obstacles confronted in the current nanotherapeutic modality [46].

The employment of the camelid-derived nanobodies in diverse areas of nanomedicine and nano therapy has contributed to the advancement of disease screening and prevention, diagnosis, treatment, and follow-up. Indeed, many commercial companies such as Ablynx, Camel-IDS, and Hybrigenics were reported to generate nanobodies [6]. In addition to their small size, camelid-based nanobodies offer exceptional properties including high sensitivity and specificity spectrum, higher safety level, water solubility and biostability, and are originally produced. These features allow nanobodies to participate significantly in the improvement of drug manufacturers and the treatment of various disorders. The tumour’s microenvironment presented a good example to demonstrate the efficient engagement of nanobodies in delivering the drug cargo to the targeted tissues. Smaller size and higher penetration capacity that portray nanobodies allow them to overcome the limitations harnessed with conventional antibody-based therapy in the treatment of solid tumours. Numerous studies including experimental preclinical investigations and clinical trials have been conducted to examine the effect of camelid-based nanobodies in the management of different diseases such as breast and lung cancers, infectious diseases, and inflammatory conditions [46].

### Involvement of camels’ biological secretions in remedying diverse health conditions

#### Camels milk components and biological proprieties

Camels’ milk has a high nutritional value [50] as it consists of numerous proteins, fats, oligosaccharides (lactose), nucleotides, essential amino acids, vitamins, and minerals [51]. Certainly, camel milk is enriched with amply endowed components including vitamin C and E, lactic acid bacteria (LAB), caseins (α, β and κ isoforms), and plenty of the whey acidic proteins such as lysozyme, alpha-lactalbumin, immunoglobulin, lactoferrin, and lactoperoxidase [52]. Additionally, different camel milk phospholipids have been also detected such as phosphatidyl-ethanolamine (PE), phosphatidyl-choline (PC), lysophosphatidylcholine (LPC), and phosphatidylinositol (PI) [53].

The highest milk production is found in the Majaheem breed (of C. dromedarius species) however there is a significant difference in the milk components among the C. dromedarius dairy camel breeds. For example, milk produced by the Sofor camel is higher in protein, fat, and lactose than that produced by other camel breeds in SA [54]. On the other hand, the milk from the Maghateer breed contains a high quantity of potassium and calcium [54]. An analytical study conducted by Salmen et al. compared the camel milk casein components profile of different camel breeds including Majaheim, Wadah, and Safrah in Saudi Arabia [55]. Among these breeds, Safrah camel milk contained the highest concentration of casein proteins (67.54%) followed by Majaheim (66.26%), and Wadah (64.07%). Furthermore, the highest level of the casein ash content of the camel milk was found to be expressed in the Wadah breed (4.1%) while the Safrah breed revealed the lowest casein ash level of 2.95% of the total contents. On the other hand, the Wadah breed showed the lowest casein moisture content (6.07%) and the Safrah breed had the highest casein moisture component of 9.33% of the total content [55]. The Majaheim breed had the intermediate value among the other breeds and showed a 3.30% and 8.89% for casein ash and moisture contents, respectively. The amino acid components both essential and non-essential were relatively demonstrated similar concentrations among the investigated breeds [55]. A subsequent comparative study that described the protein and fat composition differences among various Indian camel breeds including Bikaneri, Jasialmeri, and Kachchhi has been conducted [56]. In this study, the highest protein amount was detected in the camel milk of the Kachchhi breed (4.22%), followed by Bikaneri (3.61%) and Jasialmeri (3.37%). Additionally, the fat content also displayed different values among the three breeds and thus ascribed to the level of intake nourishment, lactation status, breed type, age, and season. The Jasialmeri camels showed a 2.99% fat component while the Bikaneriand and the Kachchhi possess 2.47% and 1.95% fat content, respectively [56].

The milk proteins, particularly lactoferrin, deliver potent probiotics, antioxidant, anti-microbial along with anti-inflammatory effects [57] as shown by Table1. Anti-microbial and anti-inflammatory roles were provided by lysozyme and immunoglobulins contents of the camel milk [37]. While antioxidant’s function was attributed to the structural proprieties of the milk proteins specifically β-caseins and to their greater composition of antioxidants amino acids [58]. Importantly, these milk proteins were found to alleviate the burden of oxidative stress and the production of the damaging free oxygen radicals that harnessed with cancer’s microenvironment thus consequently suppressing cancer cells’ proliferation [59]. As well, Korashy et al. suggested that camel milk exhibits effective apoptotic ability against liver and breast cancer cells [57]. Furthermore, the active camel whey fraction (TR35) was found to display anti-tumorigenic effects in non-small lung cancer. This was accomplished through promoting in vitro cancer cells apoptosis and inhibiting tumour growth in vivo model via activation of JNK phosphorylation and suppression of P38 and STAT3 phosphorylation [39]. The tumours battling power provided by the camel milk were validated by numerous in vitro and in vivo investigations [59–63]. Exposure of different cancer cell lines such as breast cancer (BT-474), laryngeal (HE-p2), and human hepatoma (HepG2) cells to lyophilized camel milk blocked the growth and proliferation of these cells [61]. Moreover, treated the breast cancer cells MCF-7 and the colorectal cancer cells HCT 116 with the commercial camel milk induced cancer cell autophagy manifested by cell membrane deformity, intracellular vacuoles formation, elevated LC3-II/LC3-I ratio, and formation of the autophagosomes [61].

**Table 1:** Experimental studies on camel milk (CM) and urine (CU) and their active molecules

CM/CU/Ab/Active molecule	Studied models	Status	References
HCAb/VHH Camel antibodies/nanobodies (Bivalent or univalent)	BW5147 T-cell lymphoma Lewis lung carcinoma	in vitro	[71]
CM (lactoferrin)	Huh 7.5 cells HCT-116	in vitro	[180]
Camel milk (α-lactalbumin) Camel milk Camel milk (lyophilized) Camel milk (Casein)	HepG2, HeLa, Human prostate cancer cells Breast cancer MCF7 cell Breast Cancer BT-474 HepG2, HeLa	in vitro in vitro in vitro in vitro	[57] [60] [70]
Camel urine (PM 701)	Healthy humans L1210 cell A549 cell Healthy mice	*Clinical trial* in vitro*/*in vivo in vitro in vivo	[85]
Camel urine (PMF)	Healthy mice HEPG2, HCT116, U251, A549, MCF-7, leukemia Rodent lung cancer	in vivo in vitro in vivo	[181] [86]
Camel urine PMF 701 nanoparticles (Tyrosine, glycine, cysteine, arginine, hippuric and benzoic acids, ZnO, Ag, Y, Cs, and Rb)	A549 cells (human alveolar basal epithelial cell carcinoma)	in vitro	[121]
Nanobodies labelled with ^18^F	Breast cancer HER2 overexpression	in vitro*/*in vivo	[112, 113]
Anti-HER2-specific 5F7GGC Nb nanobodies radioiodinated with^131^I IB-Mal-D-GEEEK	Breast cancer HER2 overexpression	in vitro*/*in vivo	[114]
Anti-HGF nanobodies (1E2-Alb8 and 6E10-Alb8) labelled with positron emitter zirconium-89	Brain glioblastoma	in vivo	[116]
Anti-EGFR nanobodies combination modality	Brain glioblastoma	in vitro*/*in vivo	[123]
Nanobodies conjugated to PEG-liposomes multivalent system	EGFR expressing tumors	in vitro*/*in vivo	[119]
Nanobody-based targeting module (Nb-based TM)	EGFR expressing tumors	in vitro*/*in vivo	[120]
Nb6 and Nb17 nanobodies	Pulmonary disease	in vivo	[122]

The therapeutic ability of camel milk can be delivered by its gifted biological components such as vitamin E and C [64], casein, lactoferrins, lactoperoxidase, and fatty acids, several ions and metals, and immunoglobulins [65]. It was postulated that lactoferrin delivers the majority of antitumorigenic effects of the camel milk [59, 61, 62, 66]. Lactoferrin, the principal iron-binding glycoprotein of camel milk, exerts an antioxidant influence and was found to induce in vitro anti-proliferative capacity against colon cancer cells (HCT-116) and prevent DNA damage [59]. Additionally, lactoferrin produces an inhibitory effect of cytochrome P4501A1 causing murine melanoma cancer cells (B160F10) death [62]. Furthermore, camel milk shows a high concentration level of vitamin C that revealed protection against mutagenesis and clastogenic influences [67]. Selenium and zinc elements [68], and casein [69] play a pivotal role in eliminating certain genotoxic effects of the toxic compounds and ensuring the correct synthesis of DNA and RNA thus preventing cancer development [68]. Indeed, camel milk endowed with casein containing α-lactalbumin induces cellular death and activates the apoptosis pathway in HepG2 and HeLa cells [70]. Nonetheless, the exact active components of the camel milk that specifically exert the tumour suppression effects and their downstream molecular pathways required further investigations.

Moreover, camel milk expressed distinctive immunoglobulins characterized as VHH antibodies or nanobodies [20]. These immunoglobulins are featured by their small size, unlike human IgGs, giving them the ability for tissue penetration and intracellular localization and functioning [53]. Additionally, VHH nanobodies with their biophysical proprieties can easily target the tumour tissues and the metastatic loci [71–73]. Cortes-Retamozo et al.’s investigated nanobodies in camel milk and were able to provide insight into the structural arrangements of the secreted nanobodies. They demonstrated that nanobodies are naturally occurring single-domain antigen-binding units and are less hydrophilic than the camel variable domain of HCAbs (VHH) domains. Additionally, they possess nonimmunogenic fragments and show potent specificity for targeting the solid tumours [71]. These features assist in the production of functional and stable antibody constructs with excellent target specificity against cancer cells and have been suggested as therapeutic or diagnostic tools [71]. Individual studies have reported the promising use of naturally secreted nanobodies in camels’ biological fluids, including urine and milk, as therapeutic agents for the treatment of various disorders such as chronic hepatitis and hepatitis C virus infection (HCV), peptic ulcers, inflammatory and infectious conditions, and different types of cancers [60, 74–76].

**Fig. 3:**
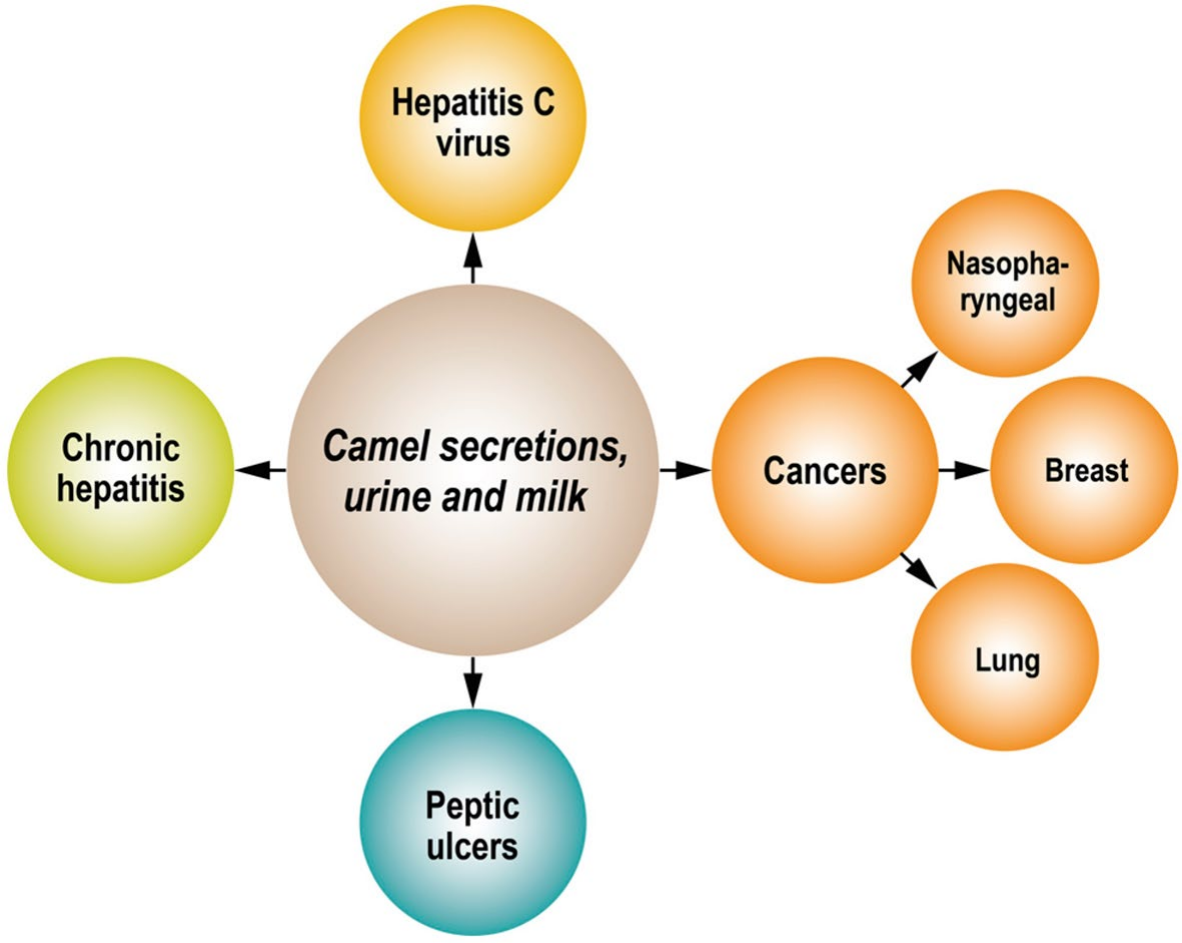
The effect of camel’s secretion on different diseases

To complete the picture, we investigated the correlation between the expression of the camel milk protein components combined and the overall survival (OS) of cancer patients with different tumour types using the online KM plotter software (http://kmplot.com/analysis/) [77]. This tool analyses the effect of 54,675 genes on the survival outcome of cancer patients using 10,293 cancer samples of different types. The genes expression signature (consists of beta-caseins (CSN2), lysozyme (LYZ), alpha-lactalbumin (LALBA), lactoferrin (LTF), and lactoperoxidase (LPO)) was obtained using the multigene classifier and the mean expression levels of all selected genes, auto select best cut off value, and ten years follow up period with HR of 95% confidence. As displayed in Fig. 3, high expression of the genes signature is associated with a significant prolongation of OS of patients with breast, cervical, head & neck, renal, sarcoma, and lung cancers confirming favourable patient outcomes. We also showed a marginally significant correlation between elevated expression of the genes signature and patients’ survival with rectal, ovarian, uterine, and bladder cancers.

#### Camels’ urine components and their biological proprieties

Despite being a waste product, camel urine has been utilized as a foundation for several therapeutic mediators. In alignment with the previous reports, a study conducted by Abdul Qader et al. showed that camel urine consists of more than 32 proteins including alpha-1B-glycoprotein, alpha-1-acid glycoprotein, serotransferrin, vitamin D-binding protein, serum albumin, and thyroxine-binding globulin. These proteins could explain the anti-inflammatory and anti-infectious effects delivered by camel urine [78]. In vitro studies suggested that camel milk and urine can inhibit mutagenesis and the proliferation of mutant cells and can induce apoptosis in cancerous cells [79]. Romli et al. analyzed the effects of nanobodies-containing camel urine on breast cancer cells (4T1) in vitro and in vivo. They found that exposure to purified camel urine in vitro inhibited the growth of 4T1 cancer cells and restricted the metastatic process of these cells. In a double-blind assessment, mice inoculated with 4T1 cells treated with camel urine showed that the size of the tumour was significantly reduced in the treated group compared to the control group [80]. Likewise, two independent studies by Evers et al. [81] and Alebie et al. [82] showed the therapeutic potential of camel milk and urine as anti-neoplastic agents [83, 84].

**Fig. 4:**
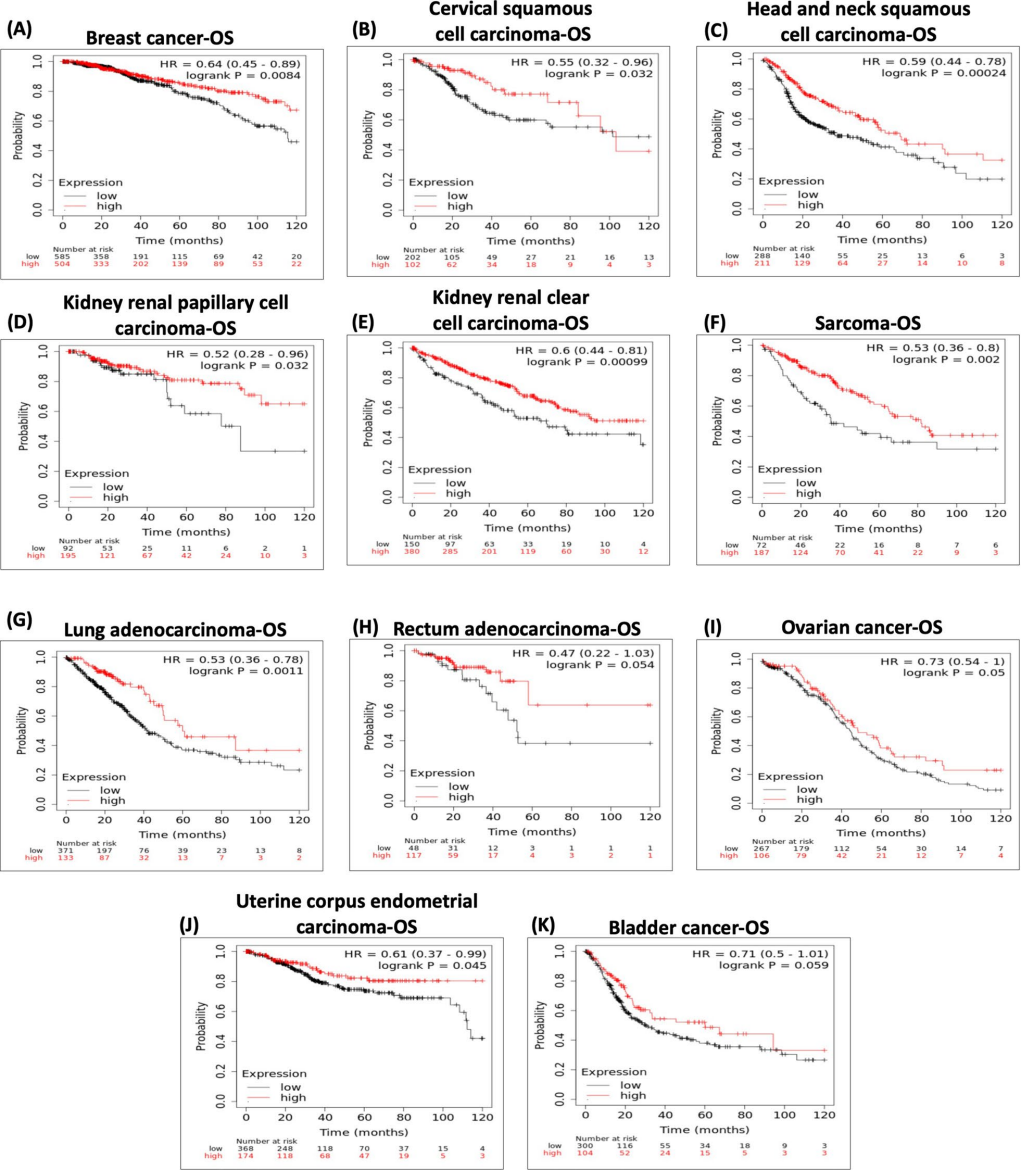
The impact of Camel’s secretion on overall survival in several diseases

Moreover, camels’ secretions, including milk and urine, revealed favourable results as a therapeutic modality in the hepatoma [70], breast cancer [57], and lungs cancer [85] (Fig. 4). In 2006, Khorshid and Moshref carried out in vitro experiments that demonstrated the ability of lyophilized camel urine to inhibit the growth of tumour cells in different cancer cell lines: hepatocellular carcinoma (HEPG2), colon cancer, lung cancer, and leukemia [85]. Khorshid and Moshref hypothesized that the anticancer action of camel urine occurred via both direct cell cytotoxicity and anti-angiogenic action (reduction of blood flow to tumour cells) [86]. The cytotoxic effect of the camel urine was further evaluated by individual reports that demonstrated the proliferative inhibitory effects and cellular cytotoxicity following camel urine stimulation of different cancer cells [87, 88]. Ten types of cancer cells were subjected to lyophilized camel urine stimulation that was divided at the end of the treatment into two groups. The first group of cells including MDA-MB-231, DAOY, MED-4, and MED-13 showed over 50% cellular death, especially in the breast cancer cells MDA-MB-231where the cellular apoptosis effect was triggered by 80%. While the other group of cells, MCF 10A, HFSN-1, U2OS, MCF-7, MED-8, LoVo and HCT-116, revealed insensitivity or marginal response to the camel urine treatment [88]. The antiproliferative effects of camel urine on the MDA-MB-231 resulted from induction of cyclin-dependent kinase inhibitor p21 causing cellular senescence [87], activation of the apoptosis intrinsic pathway by reducing Bcl-2 expression, and suppression of several cancer-associated proteins including survivin, β-catenin, and cyclin D1 [88]. Thus indicating the selectivity of the cytotoxic effect of camel urine on different cell types [88]. Yet the optimal dose with fewer side effects needs to be defined experimentally and preclinically before camel urine could be proposed for cancer therapy [89]. Indeed, the age, sex, and breeds of the camels play an essential role in modulating the camel urine’s anti-proliferative properties. The proliferation capacity of lung cancer cells (A549) was measured after being treated with camel urine extracted from Magateer and Majaheem camels of different age and sex groups. The utmost cancer cellular death was observed upon administration of the male young and adult Magateer urine as compared to other breeds. Nonetheless, the biological ingredients with potential contributions to persuade this result required further investigations [82].

Likewise, mitigation of tumour angiogenesis that is delivered by the camel urine was recently confirmed by Gader and Alhaider [76]. Additionally, Alhaider et al. reported the inhibition of inflammatory angiogenesis by adding camel urine and milk into murine sponge cells. This was achieved by reducing the key components of fibrovascular tissue, VEGF expression, macrophage recruitment, TGF-beta, and several cytokines [63]. The anticancer role of camel urine was further evidenced in a report by Cyplal in which camel urine was found to cause a substantial transcriptional suppression of the gene that encodes a carcinogen-activating enzyme [88]. Moreover, camel urine was reported to induce antitumorigenic effects by reducing the expression of different cytokines promoting tumour growth such as IL-4, IL6, and IL-10 [88, 89]. Interestingly, upregulation of the chemoprotective gene expression such as Nqo1 and Gsta1 was another suggested mechanism by which camel urine promoted tumour cytotoxicity while protecting the normal cells from reactive oxygen radicles produced by chemotherapeutic agents [82]. Collectively, these findings authenticated the strong therapeutic potential of camel biological secretions in both urine and milk against cancer cell progression.

The precise function of the anticancer components in camel urine and milk remains poorly understood. The tumorigenic suppression capacity depicted by the camel urine was attributed mostly to the abundance of iron-binding lactoferrin component [62, 90–92]. Lactoferrin was reported to induce cancer inhibitory effect both in vitro and in vivo studies by inactivation of CYP1A1 in hamster buccal pouch carcinoma causing tumour regression [93]. Different mechanisms have been suggested for lactoferrin proliferative suppression effects these include activation of Fas signalling pathway upon interaction with polysaccharides ligands ensued tumour growth inhibition [94]. Moreover, lactoferrin induces cell cycle arrest at the G1 phase and stimulates the expression of cyclin-dependent kinase (CDK) inhibitor p21 cip1 protein and p27 while reducing the expression of pAkt, cyclin E, and pRb protein levels [95]. Indeed, lactoferrin by its interaction with insulin-like growth factor-binding protein-3 [96] modulates the stability of insulin-like growth factor, which is a regulator of PI3K/Akt pathway [97]. A study using GC–MS and ICP–MS analysis of the camel urine was conducted to identify the elemental component’s profile of the camel urine [98]. Numerous metabolites with a potential contribution to the biological effects of the camel urine were detected these include benzene propanoic acid derivatives, fatty acid derivatives, amino acid derivatives, sugars, prostaglandins, erythritol, melibiose, and canavanine [98]. Canavanine is an anti-metabolite of L-arginine derivatives that possess a toxic feature. Canavanine accounts for 2% of total camel urine components and is found to deliver tumour suppression effects [99]. Consequently, camel urine provides a potentially promising therapeutic tool in combatting different types of malignancies [89].

Furthermore, several forms of camel urine have existed such as Prophet Medicine Fraction (PMF) and PM701 found to be enriched in crystals, nanorods, nanoparticles, and different organic and inorganic compounds. PMF crystals constitute numerous elements such as calcium oxalate, cystine, uric acid crystals, ammonium urate, calcium phosphate, benzoic acids, glycine, alanine, and arginine. Additionally, PMF is highly concentrated in ions including Cs, Rb, K, Ca, Cd, Y, Eu, Th, and Zn [82]. Importantly, the PMF compositions play an essential role in producing cytotoxic impact against different cancer cell types by increasing the permeability of cancer cells’ membranes allowing their lysis and destruction [82]. An important element is Zn which is present in the camel urine as ZnO this when bound to the nanoparticle produces a distinctive metal oxide nanomaterial featured with cytotoxic effects [100]. This biological effect is achieved by the generation of cellular oxidative stress [101, 102] accompany by the deformation and rupture of cancer cells’ membrane [103]. Other PMF elements such, as Cs and Rb can effectively target cancer cells and stem cells by elevating their pH level and leaving normal cells unaffected [82, 104]. Camel urine PMF was found to modulate the growth functions of both normal and cancer cells via its amino acid components [105]. Tyrosine increases PMF targeting to the cancer cells while glycine and cysteine improve PMF antioxidant capacity thus strengthening the immune system [106]. Furthermore, arginine plays important role in the modulation of the immune response and recruitment of T-cells and natural killer cells against cancer cells via activation of different cytokines including IL-12 [107], IL-23, and IFN generating antitumor immune response [108].

#### Contribution of camels derived Nanobodies in nano-oncology

Nano-oncology is a field of science that combines medicine, biochemistry, and engineering and helps in establishing tumour screening, diagnosis, and therapeutic plan [109]. To achieve an accurate cancer diagnosis, especially in the early stages of the disease, the diagnostic or imaging agent needs to localize selectively into the affected area. Nanobodies with their small size and short half-life in circulation presented a precise imaging tool with higher tumour targeting and retention and a less noisy background [110, 111]. Furthermore, nanobodies bound with a diagnostic isotope such as ^18^F were successfully used in the detection of HER2 expression in breast cancer cases using PET/CT imaging technique [112, 113]. Trastuzumab is a monoclonal anti-HER2 antibody that showed favourable results in breast cancer patients overexpressing HER2 receptors. While breast cancer cases with a low or heterogenous expression of HER2 revealed partial response to trastuzumab. Anti-HER2-specific 5F7GGC Nb nanobodies that are radioiodinated with^131^I IB-Mal-D-GEEEK were produced for determining HER2 expression in breast cancer patients before trastuzumab administration. This conjugated nanobody revealed encouraging results in targeting HER2 tumours promoting receptor internalization and subsequent signals inactivation both in vitro and in mice models, (Table. 1) [114].

Besides HER2 detection, nanobodies against other growth factor receptors, that are overexpressed in various malignancies, such as EGFR1, VEGFR2, c-Met, HGF, and CXCR7 have been generated [115–118]. In aggressive types of cancer where HGF and its receptors c-Met were highly expressed, anti-HGF nanobodies (1E2-Alb8 and 6E10-Alb8) labelled with positron emitter zirconium-89 was developed to be used for in vivo detection of HGF expression. Additionally, these designated nanobodies showed therapeutic benefits demonstrated by hindrance the tumour growth in the treated mice as compared to the control group [116]. A system composed of nanobodies conjugated to PEG-liposomes was examined both in vitro and in vivo for the ability to recognize the EGFR. This system was found to exhibit an antagonistic effect on EGFR expression resulting in receptor expression internalization and downregulation and subsequent inhibition of the tumour cells proliferation [119]. Nanobody-based targeting module (Nb-based TM) is another example of a generated nanobody that targets the expression of EGFR. This effect is achieved by inducing the recruitment of the T cells selectively to EGFR overexpression cancer cells followed by destroying tumour cells both in vitro and in vivo [120].

Additionally, Gehan et al. analyzed the anticancer effects of PMF extracted from dried camel urine (PM701). They studied the consequence of adding PMF nanoparticles to a lung cancer cells line (A549) using various microscopic and spectroscopic techniques, including scanning electron microscopy. A549 cells treated with PMF showed that the PMF exhibited two modes of action against the target cells. First, these nanobodies attacked the membrane of A549 cells and changed the packing and polarity of the membrane. Second, these nanobodies selectively targeted cancer cells and transferred the PMF into the nucleus and other organelles of abnormal cells through endocytosis. In this regard, this nanobody-based drug delivery system (DDS) achieved the purpose of the smart drug treatment [121]. Similarly, Nanobodies were also used in animal studies to target the pulmonary surfactant protein A (SPA) associated with airway diseases. Nb6 and Nb17 nanobodies were developed and demonstrated rapid accumulation in the pulmonary tissues as shown by the imaging technique. This effect was accompanied by fast clearance from the circulation with a minimum build-up in the liver and spleen [122]. Moreover, the involvement of nanobodies as therapeutic agents for different brain tumours such as glioma and glioblastoma was also investigated [123]. In vitro anti-EGFR nanobodies suppressed tumour cell proliferation and growth [124]. Also, a combination modality consisting of an anti-EGFR nanobody, pro-apoptotic EGFR-specific nanobody, and an immune conjugate targeting tumour necrosis factor-related apoptosis-inducing ligand (TRAIL) displayed a potent inhibitory effect on tumour growth and aggressiveness both in vitro and in vivo [123].

The insinuation of nanobodies in medical determinations has immensely progressed as numerous nanobodies have been tested in the preclinical setting, while many of them are being examined in clinical trials as demonstrated in Table 2 [111, 125, 126]. The respiratory disease mostly benefits from the aerosol or inhaler route of administration to ensure the accumulation of the drug into the lungs and thus avoiding systemic side effects [127]. Nanobodies with their unique biological features provide an excellent vehicle for drug pulmonary delivery. ALX-0171 is a 42 kDa trivalent nanobody inhaler that is currently being tested in clinical trials (Table 2) to fight against the human respiratory syncytial virus (RSV) and shorten the duration of the infection [128]. A phase I clinical trial demonstrated the safety and tolerability of ALX-0171 in the examined individuals [127]. Caplacizumab is the first EMA and FDA-approved 28 kDa bivalent nanobody (ALX-0681) for treating patients with Thrombotic thrombocytopenic purpura (TTP). TTP is an autoimmune hematological disorder manifested by the presence of autoantibodies that target the von Willebrand factor causing platelet clumps and microvascular thrombosis and blockage. The standard management for TTP is daily plasma exchange to eliminate the autoantibodies from the circulation, in addition to immunosuppressive therapy. ALX-0681 in combination with the standard remedy for TTP displayed significant mitigation of platelet aggregation and a satisfactory safety profile, low disease burden and recurrence, and prolonged overall survival [129].

**Table 2:** Examples of nanobodies that are currently being tested in clinical trials

Nanobodies compound	Clinical use	Target	Stage	References
Caplacizumab ALX-0681 ALX-0081	Thrombotic thrombocytopenic purpura	Ultra large von Willebrand factor	Clinical trial phase III	[129]
ALX-0171	Lower respiratory tract infection	RSV (respiratory syncytial virus)	Clinical trial phase I-II	[128]
Bispecific nanobody**-**derived CAR-T cells	Lymphoma	CD19 CD20	Clinical trial phase I	[182]
[^131^I]-SGMIB anti-HER2 VHH1	Breast cancer	HER2	Ongoing	[114]

#### Effects of camel milk on modulation of various signalling pathways

As previously described the camels’ biological secretions have shown to exert therapeutic benefits in treating numerous health conditions. However, the precise subcellular molecular mechanisms that are regulated by these secretions necessitate further investigations. Camel milk was reported to induce a hypoglycemic effect in both human and animal models and was suggested as a complementary therapy in the type 1 diabetes [130–132]. To understand by which mechanism can camel milk regulate insulin signalling to produce its glycemic effect, the human embryonic kidney 293 (HEK293) cells, that expressed transiently human insulin receptors (hIR), were exposed to camel milk stimulation. Bioluminescence resonance energy transfer (BERT) assay was used to examine the activation of insulin signalling by measuring the intensity of the physical interaction between hIR and insulin receptor signalling proteins (IRS1) and the growth factor receptor-bound protein 2 (Grb2). Only simultaneous treatment with insulin and camel milk was found to significantly enhanced the intensity of BERT signals between hIR and Grb2 but not IRS1. Furthermore, camel milk was found to potentiate ERK1/2 but not Akt activation downstream insulin signalling [133, 134].

The anti-inflammatory and antioxidant effects exhibited by camel milk components were also validated in a rats model with acute respiratory distress syndrome (ARDS). ARDS is defined as the formation of significant inflammatory background causing damagethe to alveolar epithelial and endothelial barriers ending in respiratory failure [135, 136]. The animal with ARDS treated with camel milk displayed a notable reduction in the pulmonary wet: dry weight ratio, neutrophil infiltration, alveolar and interstitial edema, lung damage, and improvement in pulmonary functions. Additionally, camel milk administration alleviated the pulmonary recruitment of numerous cytokines (TNF, IL-10, and IL-1b) and oxidative stress factors. Interestingly, the observed camel milk therapeutic effects were postulated to happen through suppression of the MAPK signalling cascade [137]. Likewise, the camel milk's anti-inflammatory power was further authenticated in another animal model presented with arthritis and air pouch edema, mirroring human rheumatoid arthritis (RA) disease. RA is an autoimmune disease characterized by chronic inflammatory status causing destructive lesions in the bones and cartilages of the small joints particularly the hands [138]. In this study, the administration of camel milk resulted in the improvement of paw edema and the inflammatory features associated with arthritis. Interestingly, camel milk inhibits the phosphorylation of p38 resulting in the downregulation of MAPK, ERK1/2, and JNK1/2 cascade activities. Additionally, in comparison to the control group the treated animal demonstrated lower levels of lipid peroxidase and nitric oxide and elevated glutathione levels and antioxidants factors. Thus suggesting the potent anti-inflammatory power delivered by camel milk contents in attenuating the arthritis process [139].

Cyclosporin is an immune suppressant agent that is widely used for several autoimmune diseases. The most harmful side effects of this drug are kidney injury that can progress to renal failure. Camel milk, as an anti-inflammatory/antioxidant mediator, was proposed as a natural agent to ameliorate the side effects associated with cyclosporine administration. In vivo Administration of camel milk for 3 weeks reduced dramatically the levels of the biomarkers related to renal damage such as serum creatinine, BUN, and KIM-1. Furthermore, camel milk treatment is associated with a marked lowering in numerous inflammatory cytokines and degradation signals such as MCP-1, IL-8, TNF, MMP-2, and MMP-9. Besides the ability of the camel milk to inhibit p38/ERK/JNK MAPK signalling, camel milk blocked NF-κB pathway activation by suppressing the expression of NF-κBp65, p-NF-κBp65, and p-IκBα proteins. Curtailing of pathways together with enhancement of glutathione and antioxidant activities resulted in alleviating of the oxidative stress on the kidneys. Therefore, camel milk can be used as a natural protecting remedy against renal damage triggered by cytotoxic drugs [140].

Camel urine, the other camel’s biological secretion, was also reported to exert a tumour suppression effect and augment the doxorubicin treatment for breast cancer cells. This anticancer impact was obtained by inducing cancer cells apoptosis and DNA damage, reversing the EMT markers, and retrieving the expression of the E-cadherin epithelial marker. The author suggested the potential involvement of the camel urine component in attenuation of NF-κB-Snail signalling and its downstream inflammatory mediators thus abrogating breast cancer cells' aggressive phenotype [141].

## Nano proteomics

The camel genome sequencing project was completed in 2003, and the data it produced gave researchers the chance to use proteomics to analyze and gain insight into all of the cellular processes that could not be explained by genomics alone. A proteomics study offers helpful information on the post-translational modifications, localizations, structures, and interactions of all proteins expressed by an organism in addition to merely studying the entire proteome [142]. Also, proteomics has different clinical applications which include helping researchers evaluate the safety and efficiency of therapeutic interventions [143, 144]. Examining the processes underlying drug responses, discovering new therapeutic targets, understanding the etiology of disease, finding new biomarkers for disease early detection [145, 146]. Functionally, biomarkers are classified into three major categories: biomarkers for early disease detection, prognostic biomarkers that provide information about the potential for malignancy, and predictive biomarkers that distinguish between various cancers to provide an appropriate therapy [147].

The immense challenge of finding minute quantities of biomarkers from natural fluids such as urine, saliva and blood, compared to the simpler detection of high-abundance proteins, has led to the emergence of a new field called nano proteomics, which integrates proteomics and nano techniques [148–150]. Nano proteomics has been utilized in the discovery of biomarkers in two main areas: nanostructured surfaces and nanoporous materials. Several nanomaterials including gold nanoparticles, quantum dots, nanowires and carbon nanotubes have been developed to help researchers overcome the challenges of detecting low-abundance proteins [151, 152]. Nano proteomics provides a sensitive and robust analytical platform for screening low-abundance biomarkers in a high-throughput manner [150]. Of note, nano proteomics has been used to detect biomarkers associated with various human diseases, including autoimmune diseases [153] and cardiovascular disorders [154]. Therefore, the main concern of cancer researchers is being able to use nano proteomics to facilitate prompt and safe cancer diagnosis. Consequently, various analyses have been conducted on select biomarkers in distinctive types of cancer, including breast and prostate cancers [155, 156]. However, the compatibility and toxicity of nano techniques remain a health concern and further research needs to be implemented to guarantee their safety in biological applications [157].

The exceptional pharmacokinetic and physicochemical properties of camel nanobodies are consistent with those needed for cancer therapy and provide added benefits over conventional antibody technology in drug delivery, immunotherapy, and diagnostics. The affinities of camel-heavy antibodies are higher than those of conventional antibodies and can reach up to 100 pM affinity constants [158, 159]. This facilitates protein-protein interactions and can act as a strong basis for identifying cancer biomarkers and intracellular signalling. Heavy-chain antibodies can also be bonded with fluorescent dyes to generate chrome bodies that can be used in single-molecule localization with super-resolution imaging techniques [160–162]. The presence of cysteines at amino acid positions 54 and 78 makes a more stable disulphide bond at the hydrophobic region [163, 164]. Additionally, nanobodies are very robust as their melting points are in the range of 67 °C–78 °C, with the ability to refold after thermal unfolding has been detected and with functional activity up to 90 °C [18]. The robustness of nanobodies (heavy-chain antibodies) makes them very suitable for the antibody engineering [165, 166], tumour targeting [8], and pharmaceutical formulations [167]. Moreover, nanobodies can distinguish recessed antigenic domain, a property that has been attributed to their small size and the capacity of the prolonged CDR3 loop to rapidly infiltrate the epitopes [159, 168]. Thus, nanobodies can be successfully applied to target some enzymes or transmembrane proteins or even signalling pathways in certain tumour cells. This was demonstrated by Cortez-Retamozo et al. in 2004 who targeted tumour cells through the nanobodies fused to the β-lactamase enzyme to identify the carcinoembryonic antigen [169]. The enzyme converts an injected non-toxic pro-drug into a toxic drug with an elevated concentration in the targeted tumour cells. Such like nanobody conjugates hold much promise for cancer immunotherapy. It has been well established that cell-surface protein conjugates used to target the epidermal growth factor receptor can hinder the epidermal growth factor by binding to its receptor, this technique has been used to cure solid tumours [124]. Similarly, nanobodies targeting the tumour necrosis factor-α (TNF) could be applied to cure malignant tumours [170].

## Bioinformatics and nanobodies

Advancements in computational molecular biology, such as the formation of large genomic and proteomic databases and the development of various tools and bioinformatic software, facilitated the achievement of various benchmarks that can be used for therapeutic purposes. Bioinformatics is playing a vital role in understanding complex metabolic pathways and discovering various vital components of living systems [171]. Several bioinformatic platforms such as MATLAB Symbiology and Pathway Studio, amongst others, have proven useful in studying and identifying various nanobodies. These bioinformatic tools have been used in an integrated method for graph-based visualization of the components of complex networks. Such bioinformatics systems use literature and microarray databases of nanobodies to identify novel nanobodies that are involved in genetic regulatory pathways. This strategy has revealed novel interactions among nanoparticles and genes, providing valuable details that contribute to the current understanding of the various nanoparticles that are involved in complex cellular pathways. Also, the approach presents an advanced research platform to find nanoparticles that can be used in DDS for therapeutic purposes and to advance the bio- and chemo-informatics analyses of molecular pharmacology.

Research has also been carried out to utilize and explore the combined potential of nanoparticles and bioinformatic tools for the treatment of cancer [172]. In 2012, Arvizo and colleagues reported that integrated bioinformatics, proteomics, and nanotechnology strategy could be used to discover unique therapeutic targets for cancer by proposing that protein corona are formed when proteins bind to nanoparticles [172]. Using corona with engineered surface-functionalized gold nanoparticles (AuNPs) revealed proteins that provided insight into the development and stages of ovarian cancer. They concluded that protein corona modulation around nanoparticles is a promising therapeutic approach for various diseases, including ovarian cancer. Given the advancement in animal biotechnologies, including camel cloning and transgenesis, the design of targeted nanobodies secreted from genetically engineered camels would be specific tools to treat tumours. Recent reports have revealed the possibility of delivering anti-EGFR therapies to brain tumours through the invention of stem cell-delivered anti-EGFR nanobodies. These inhibited tumour cells when combined with cytotoxic molecules, considerably enhancing therapeutic outcome [123]. More so, the isolation of camel stem cells was recently achieved by our group [173] which could further improve the efficiency of camel cloning and transgenesis and thus provide a versatile model for generating targeted nanobodies for treating cancer cells [123].

## Exosomes

Exosomes are nano-sized extracellular membrane vesicles that range in size from 50 to 200 nm and are unique nanoparticle drug carriers with important nanoparticle features [174, 175]. Exosome biomimetic nanoparticles are advantageous in their ability to incorporate both synthetic and natural materials to create a more efficient drug delivery method. Yet they still have limitations due to the method of synthesis, which is reflected in the protein integrity on the exosome surfaces, which compromises their functioning [176]. Natural sources of exosomes from biological fluids, such as milk, are therefore important, especially if we are aiming for production in a large-scale [74, 177].

Preliminary studies have shown that camel milk exosomes could play an important role in inhibiting the growth of breast cancer cells [178]. They could also modulate cyclophosphamide-induced oxidative stress and immunotoxicity in mammals. These effects stem from the importance of biomolecules carried by exosomes, such as bioactive lipids, a specialized functional proteome, nucleic acids (including DNA, microRNA and ncRNA), metabolites, and signalling molecules that can be transported over distance within the protection of a lipid bilayer–enclosed structure [179].

Furthermore, EL-Kattawy et al. found that camel colostrum-derived exosomes, that are enriched in milk protein contents, showed remarkable apoptotic effects on liver cancer cells exclusively sparing the normal cells unaffected. These carcinogenic suppression effects are attained by elevation in the expression level of both Bax and caspase3 and reduction in Bcl2 level. This was accompanied by a reduced expression level of the inflammatory mediators such as TNFα, NFkB, TGFβ1, and Cox2 as well as the angiogenic-related factors VEGF [36].

## Concluding remarks and perspectives

In this review, we discussed the therapeutic value of the camelid-derived nanobodies along with the therapeutic effects provided by the ingredients of camels’ biological secretion both milk and urine in combatting different human diseases mostly cancers. Many research areas such as medical, biotechnology, engineering, and economics have been cultivated after the discovery of the naturally occurring camelids nanobodies. Nanobodies have recently emerged as attractive robust strategies for diagnostic and therapeutic aims, particularly in the cancer field. Providentially, nanobodies exhibit several biophysical and biochemical proprieties besides the ability to cross the biological membranes such as the blood-brain barrier. Furthermore, they show the capacity to enter smoothly into solid organs including the brain, lymph nodes, lungs, and liver. Because nanobodies presented flexible manufacturing and assembly they have been incorporating into multipotent constructs or conjugating with chemotherapeutic drugs to produce highly specific and efficient compounds. These features make nanobody-based therapies useful and powerful tools to deliver the drug into the targeted tissues exclusively. Nevertheless, nanobodies still displayed some pitfalls due to their minute sizes including rapid renal clearance and a possibility of renal toxicity. This drawback can be modified by coupling nanobodies with the serum albumin to increase their retention time in the circulation, yet such an approach will inexorably reduce their diffusion and penetration benefits. Further investigation is pivotal to elucidate their exact molecular mechanisms in modulating oncogenic signalling pathways and to assess their safety profile in preclinical experimental studies before the implication in any clinical trials.

## Data Availability

Data sharing is not applicable.

## Abbreviations

A549: Lung cancer cell line
BGI: Beijing genomics institute
C. bactrianus: Camelus bactrianus
C. dromedaries: Camelus dromedarius
CM: Camel milk
DDS: Drug delivery systems
HCV: Hepatitis C virus
IARC: International agency for research on cancer
KACST: King Abdulaziz city for science and technology
N/A: Not applicable
NCGT: National center for genomic technology
PMF: Prophet medicine fraction
PRP: Peptidoglycan recognition protein
SA: Saudi Arabia
TNF: Tumor necrosis factor-Α
VHH: Variable domain of HC antibodies
