# Correction: Camels’ biological fluids contained nanobodies: promising avenue in cancer therapy

**DOI:** 10.1186/s12935-023-02898-7

**Published:** 2023-03-30

**Authors:** Nouf S. Al‑Numair, Abdulrahman Theyab, Faisal Alzahrani, Anwar M. Shams, Ibrahim O. Al‑Anazi, Atif Abdulwahab A. Oyouni, Osama M. Al‑Amer, Charalampos Mavromatis, Islam M. Saadeldin, Wed A. Abdali, Yousef M. Hawsawi

**Affiliations:** 1 Center of Genomic Medicine, King Faisal Specialist Hospital & Research Center, Riyadh, Saudi Arabia; 2 College of Medicine, Alfaisal University, P.O. Box 50927, Riyadh, 11533 Saudi Arabia; 3 Department of Laboratory & Blood Bank, Security Forces Hospital, P.O. Box 14799, Mecca, 21955 Saudi Arabia; 4 College of Medicine, Al-Faisal University, P.O. Box 50927, Riyadh, 11533 Saudi Arabia; 5 Department of Biochemistry, Faculty of Science, Embryonic Stem Cells Unit, King Fahad Medical Center, King Abdulaziz University, Jeddah, Saudi Arabia; 6 Centre of Artifcial Intelligence in Precision Medicines (CAIPM), King Abdulaziz University, Jeddah, Saudi Arabia; 7 Department of Pharmacology, College of Medicine, Taif University, P.O. BOX 11099, Taif, 21944 Saudi Arabia; 8 The National Center for Genomic Technology, King Abdulaziz City for Science and Technology, P.O Box 6086, Riyadh, 11442 Saudi Arabia; 9 Department of Biology, Faculty of Sciences, University of Tabuk, Tabuk, Saudi Arabia; 10 Genome and Biotechnology Unit, Faculty of Sciences, University of Tabuk, Tabuk, Saudi Arabia; 11 Department of Medical Laboratory Technology, Faculty of Applied Medical Sciences, University of Tabuk, Tabuk, Saudi Arabia; 12 Department of Biological Sciences, Faculty of Science and Arts (Rabigh Campus), King Abdulaziz University, Jeddah, Saudi Arabia; 13 Research Institute of Veterinary Medicine, Chungam National University, Daejeon, 34134 Korea; 14 Research Center, King Faisal Specialist Hospital and Research Center, MBCJ04, PO Box 40047, Jeddah, 21499 Saudi Arabia

**Correction: Cancer Cell International (2022) 22:279** **https://doi.org/10.1186/s12935-022-02696-7**

In this article, the affiliation details for the author Prof. Yousef M. Hawsawi were incorrectly given as “2,3,4,5,6,7,8,9,10,11,12,13,14” but should have been “2, 14”.

The original article [1] has been corrected.
